# Reorganization of Intact Descending Motor Circuits to Replace Lost Connections After Injury

**DOI:** 10.1007/s13311-016-0422-x

**Published:** 2016-02-03

**Authors:** Kathren L. Fink, William B. J. Cafferty

**Affiliations:** Department of Neurology, Yale University School of Medicine, New Haven, CT 06520 USA

**Keywords:** Spinal cord injury, Plasticity, Regeneration, Repair, Axon, Neuron

## Abstract

Neurons have a limited capacity to regenerate in the adult central nervous system (CNS). The inability of damaged axons to re-establish original circuits results in permanent functional impairment after spinal cord injury (SCI). Despite abortive regeneration of axotomized CNS neurons, limited spontaneous recovery of motor function emerges after partial SCI in humans and experimental rodent models of SCI. It is hypothesized that this spontaneous functional recovery is the result of the reorganization of descending motor pathways spared by the injury, suggesting that plasticity of intact circuits is a potent alternative conduit to enhance functional recovery after SCI. In support of this hypothesis, several studies have shown that after unilateral corticospinal tract (CST) lesion (unilateral pyramidotomy), the intact CST functionally sprouts into the denervated side of the spinal cord. Furthermore, pharmacologic and genetic methods that enhance the intrinsic growth capacity of adult neurons or block extracellular growth inhibitors are effective at significantly enhancing intact CST reorganization and recovery of motor function. Owing to its importance in controlling fine motor behavior in primates, the CST is the most widely studied descending motor pathway; however, additional studies in rodents have shown that plasticity within other spared descending motor pathways, including the rubrospinal tract, raphespinal tract, and reticulospinal tract, can also result in restoration of function after incomplete SCI. Identifying the molecular mechanisms that drive plasticity within intact circuits is crucial in developing novel, potent, and specific therapeutics to restore function after SCI. In this review we discuss the evidence supporting a focus on exploring the capacity of intact motor circuits to functionally repair the damaged CNS after SCI.

**Electronic supplementary material:**

The online version of this article (doi:10.1007/s13311-016-0422-x) contains supplementary material, which is available to authorized users.

## Introduction

Our ability to execute complex motor functions is afforded by the intricate integration of multiple descending motor pathways synapsing upon spinal interneurons and motor neurons that ultimately activate target muscle groups. This complexity comes at a price, as trauma due to spinal cord injury (SCI) interrupts these pathways and results in a permanent loss of normal function as original axonal connections fail to regenerate. Therefore, the functional prognosis for patients with SCI is currently limited. However, while full recovery is rare, a modest amount of spontaneous recovery is observed acutely after trauma that is titrated by the extent and location of the injury, in patients and rodent models of SCI [1, 2]. The structural and molecular mechanisms that mediate spontaneous functional recovery are unknown and hence our inabilities to exploit them represent a significant barrier to therapeutic design. In this review, we explore the evidence supporting a role for uninjured motor circuit plasticity in supporting spontaneous functional recovery after SCI.

## Wiring of Descending Motor Systems

Dissecting the potential for intact motor circuit rearrangement to restore function after experimental SCI requires detailed knowledge of the spinal fasciculation and termination pattern of the major descending pathways in the intact adult. Careful anatomical mapping of the major descending motor pathways has been achieved via delivery of extrinsic tracers and the engineering of specific transgenic reporter lines [8–11]. These studies have revealed the detailed spinal termination pattern of each of the major descending motor pathways. The same approaches have also been used to determine whether structural rearrangements of these fiber tracts occur after experimental SCI. To facilitate comparative anatomical analysis of motor pathways before and after SCI, investigators have focused on the 4 major motor centers described in detail by Kuypers and colleagues [12], who grouped motor pathways based on their spinal termination patterns.

**Fig. 1:**
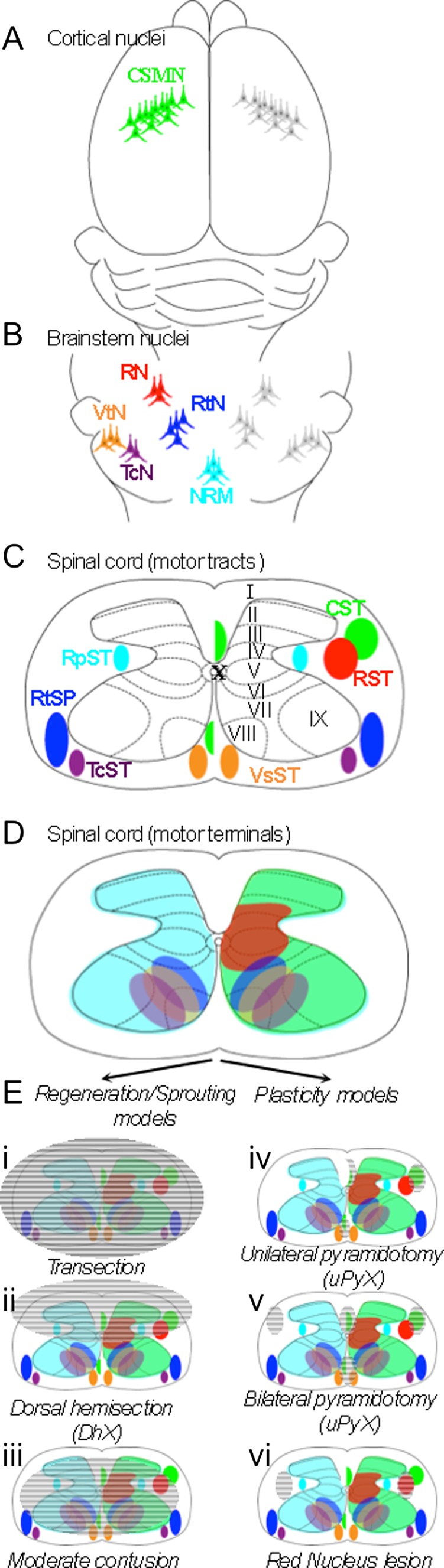
Schematic organization of descending motor tracts. (**A**) Bilateral location of corticospinal motor neurons (CSMNs) in layer V of sensorimotor cortex, the somata of which give rise to the corticospinal (CST) and corticofugal projections. Green CSMNs are highlighted to represent the descending fasciculated spinal course (**C**) and termination pattern of one side of the CST projection. (**B**) Location of motor center brainstem nuclei including the red nucleus (RN; red), medullary reticular nuclei (RtN; blue), vestibular nuclei (VtN; orange), tectospinal nuclei (TcN; purple), and the nucleus raphe magnus (NRM; cyan). These nuclei give rise to (**C**) the rubrospinal tract (RST; red), the reticulospinal tract (RtST; blue), the vestibulospinal tract (VtST; orange), tectospinal tract (TcST; purple) and the raphespinal tract (RpST; cyan). One side of the fasciculated projections of these pathways are schematized in (**C**) and their terminals within spinal gray matter in (**D**). Note extensive overlap of motor terminal distribution in the spinal ventral horn. Lesion models to study regeneration and sprouting of lesioned axons are schematized in (**E**i–iii). The transection model completely interrupts all descending projections [hatched lines in (i)], the dorsal hemisection (DhX) model lesions the dorsal half of the spinal cord (ii) interrupting the CST and RST, leaving the VtST, TcST, and RtST intact. (iii) Moderate thoracic contusion destroys the central core of the spinal cord at the lesion epicenter, thus ablating spinal gray matter entirely and sparing a limited amount of all circumferential descending motor pathways. (**E**iv–vi) Lesion models to study plasticity of intact motor pathways. (iv) The unilateral pyramidotomy (uPyX) model lesions 1 side of the CST in the brainstem, thereby sparing the contralateral CST and all other descending motor pathways. (v) Bilateral pyramidotomy (bPyX) lesions both sides of the CST, sparing all other descending motor tracts. (vi) RN lesion specifically ablates the RST, sparing all other descending motor pathways

The first group includes the “corticobulbar and corticospinal pathways”. The somata of corticobulbar pathways reside in Layer V of cortex and terminate diffusely upon multiple brainstem nuclei. The cell bodies of corticospinal tract (CST) motor neurons also reside in Layer V (Fig. 1A); however, they project their axons through the brainstem, decussate in the caudal medulla, and descend predominantly in the ventral dorsal columns in the rodent (Fig. 1C), and lateral columns in humans and primates [13]. CST axons terminate unilaterally throughout every segment and lamina of spinal gray matter (Fig. 1D). The second division includes the “group A brainstem pathways”, which comprises the vestibulospinal, reticulospinal (RtST), and tectospinal tracts (Fig. 1C). The somata of these tracts reside in the ventromedial portions of the brainstem, project down the spinal cord in the ventrolateral funiculi and terminate bilaterally in the ventromedial spinal grey matter (Fig. 1B,D). The third division includes the “group B brainstem pathways”, which comprise the rubrospinal (RST) and pontospinal tracts (Fig. 1B). The RST arises from the magnocellular region of the red nucleus and the pontospinal pathway from the ventrolateral pontine tegmentum. The axons of these pathways descend in contralateral white matter and innervate intermediate spinal gray matter (Fig. 1C,D). The final group is the “emotional centers of the brainstem”, which includes groups of fiber tracts originating from the nucleus raphe magnus (NRM; Fig. 1B), the locus coeruleus, and subcoeruleus. The best described is the raphespinal tract (RpST), which descends in the intermediolateral spinal columns and terminates diffusely throughout every segment of spinal gray matter (Fig. 1C,D). The division of motor centers dictated by spinal termination pattern described by Kuypers et al. shows that extensive overlap exists between the terminals of many motor pathways (Fig. 1D). This has been confirmed in studies that have completed selective elimination of single descending motor tracts and observed only minor or task-specific deficits without impairing gross motor function [14–17]. Thus, extensive functional redundancy between descending motor tracts suggests that intact circuit re-arrangement could functionally compensate after injury. To investigate the capacity of intact circuit rearrangement to drive functional recovery after injury, investigators have sought specific lesion models to parse the functional implications of growth from lesioned and/or intact axons after partial experimental SCI.

## Models to Study Axon Regeneration

Clinically, SCI results in permanent functional deficits because neurons have a limited capacity to grow in the adult central nervous system (CNS). Anatomically, there are two broad routes to achieving restitution of functional circuitry, either long-distance regeneration of cut axons or, alternatively, plasticity and/or sprouting of lesioned and intact axons [18, 19]. Emerging data suggest both mechanisms respond to pro-axon growth experimental interventions (discussed below). Achieving functional restitution via regeneration requires that damaged axons survive axotomy, initiate and maintain a growth response over long distance, respond to appropriate guidance cues, circumvent areas of glial scarring and degenerating myelinated axonal profiles, and finally, form functional synapses. To date, utility of even the most potent combinatorial therapies has resulted in limited axonal regeneration yet modest functional recovery after experimental SCI [20–22]. The absence of long-distance regeneration clearly demonstrates that alternative anatomical mechanisms support functional recovery. Identifying the anatomical mechanisms supporting spontaneous and intervention-driven functional recovery are crucial to designing novel potent therapeutic interventions. To this end, recent studies have directly shown that axotomized neurons sprout over a short distance to make new local synaptic connections with short and long propriospinal circuits, thus circumventing lesion sites and obviating the need for long distance axon growth [9, 23, 24]. Additionally, sprouting of intact axons within the spinal cord and within motor centers of the brainstem can utilize the same “bypass circuit” mechanisms to either drive activity within original denervated targets or utilize remaining intrinsic propriospinal circuitry to bridge structures across lesioned areas [17, 25–27]. To differentiate between the functional impacts of axon growth between intact and axotomized neurons, investigators have utilized a variety of lesion models ranging in severity and thus percentage and identity of spared pathways. These models fit into two categories: first, those that lesion multiple descending pathways, and therefore focus on regeneration and sprouting of axotomized neurons (Fig. 1Ei–iii); and second, those that interrupt single descending tracts and focus on plasticity of remaining intact pathways (Fig. 1Eiv–vi).

Common lesion models that interrupt multiple descending spinal tracts include transection, hemisection, and contusion. Spinal transection represents the most severe experimental SCI (Fig. 1Ei). In this model, all connections between the proximal and distal portions of the spinal cord are eliminated, resulting in flaccid hindlimb paralysis in rodent models [28]. Therefore, regeneration of damaged axons across the injury site is absolutely required for functional improvement. Few studies have shown functional improvement after deploying this model [28]. Therefore, investigators more commonly use less severe, subcomplete injury models that spare a varying portion of the spinal cord. These models retain a conduit to allow for a combination of regeneration of cut axons and sprouting or plasticity of intact axons to occur that could support functional recovery. The 2 most frequently used models of incomplete SCI include the thoracic dorsal hemisection (DhX) lesion and the thoracic contusion lesion (Fig. 1Eii,iii). Both of these models allow for a near complete elimination of the primary descending motor tract in mammals, the CST [13]. Therefore, anterograde tracing of regenerating CST axons with biotin dextran amine (BDA) is commonly used as the primary anatomical outcome measure in these investigations [8]. These models differ, however, in the amount of spared tissue that remains after injury. After DhX, spinal tissue ventral to the central canal is entirely spared; therefore, any functional improvement after systemic therapeutic intervention (pharmacological or constitutive knockout) could result from either regeneration of the cut dorsal CST or sprouting of intact tracts in the ventral spinal cord, including the ventral CST or the RtST [9, 29]. Mid-thoracic contusion injury is less selective with regard to sparing as this lesion typically results in complete destruction of gray matter at the lesion epicenter and sparing only of a circumferential ring of intact white matter [30]. Far fewer spared axons are capable of driving functional recovery, thus rendering the contusion model unsuitable for studying the relative contribution of regeneration of lesioned axons and sprouting of intact axons in promoting functional recovery after SCI. Both DhX and contusion lesion result in reproducible hindlimb paralysis, recovery from which can be measured using standardized open-field locomotion scores [30–32]. Therefore, these subcomplete lesion models are extremely valuable for studying regeneration-associated functional recovery.

## Models to Study Structural Plasticity

While complete transection, DhX, and contusion injury result in reproducible functional deficits, identification of the anatomic pathways that support spontaneous or therapy-induced functional recovery is complicated by an inability to differentiate between the impacts of axon growth from lesioned and/or intact pathways. To this end, investigators have sought subtler lesion models that can directly assess the functional impact of structural plasticity of intact spinal circuitry. These approaches have focused on selective lesions of 1 specific descending motor pathway that results in reproducible functional deficits that incompletely resolve over time. The best studied is the pyramidotomy (PyX) lesion model. In this model, the CST is selectively lesioned in the brainstem either unilaterally (uPyX; Fig. 1Eiv) or bilaterally (bPyX; Fig. 1Ev). Owing to the lesion site residing in the brainstem, regeneration of cut CST axons after PyX is unlikely to reach the spinal cord and make significant functional connections. Therefore, singular ablation of the CST allows for plasticity and/or sprouting of intact tracts, either the intact CST after uPyX or other descending motor tracts after bPyX, to be studied. PyX results in reproducible behavioral deficits in forelimb pellet retrieval, grid-walking, tape removal, and spontaneous vertical exploration that can be assessed over time to ascertain whether a therapeutic intervention that enhances growth of intact axons translates into functional improvement [33]. Other descending motor pathways have proved more difficult to ablate specifically, owing to their physical location deep within spinal white matter. However, selective ablation of the RST is possible via electrolytic lesion of RST somata in the red nucleus (Fig. 1Evi). This procedure has revealed that the RST is functionally redundant when the CST is intact [34]. However, RST function is critical after CST ablation, further highlighting the necessity for selective lesion studies in dissecting functional recovery after partial SCI.

In developing strategies to enhance regeneration or sprouting after SCI, it is important to remember that pro-axon growth therapies will affect both damaged and intact neurons. This may be detrimental in the incidence of maladaptive plasticity of primary afferent sensory neurons, precipitating the emergence of neuropathic pain [35, 36]. However, it may also be beneficial in achieving greater functional recovery than what would be possible if only CST regeneration occurred. Moving forward, the sensitivity of different neuronal populations to pro-axon growth strategies is a critical consideration to focus therapeutic design.

In experimental SCI models where limited to no regeneration has been seen, there is still spontaneous recovery of function. It is hypothesized that the locus of this functional recovery is due to the reorganization of intact circuits rostral and caudal to the lesion site. Understanding how circuits reorganize and the extent to which this non-regeneration approach to repairing the spinal cord is able to restore function will refine the development of therapeutics to treat patients with SCI. For the remainder of this review we will discuss the evidence supporting spontaneous reorganization of intact circuits with particular focus upon the CST, RST, RpST, and RtST, and the molecular interventions that have been shown to enhance spontaneous plasticity of these pathways to restore motor function.

## Molecular Brakes on Plasticity After SCI

Damaged axons fail to regenerate after SCI owing to the low intrinsic growth capacity of adult CNS neurons and the inhibitory axon growth environment in the mature CNS [37, 38]. The intrinsic factors that promote growth of CNS neurons during development and the early postnatal period are beginning to emerge. It has been hypothesized that at least during development of the CST, differential cyclic nucleotide monophosphate levels may drive axon growth in response to gradients of guidance cues [13]. Recent reports assessing regeneration of retinal ganglion cells after optic nerve lesion suggest that only retinal ganglion cell subtypes with high intrinsic activity of mammalian target of rapamycin (mTOR) signaling are capable of regenerating after lesion [39]. Targeting the mTOR pathway, via inhibition of phosphatase and tensin homolog (PTEN) or through constitutively active Rheb, has produced robust axon regeneration and plasticity after SCI but with no reported functional benefits without combinatorial treatment [5, 40]. However, inhibition of PTEN may not be a clinically viable therapy owing to its potentially carcinogenic side effects [41]. Additional studies that have sought to identify other activators of axon growth using genetic screens have identified *dlk1*, *klf7*, and *sox11* as critical factors in setting the intrinsic growth capacity of developing neurons [3, 42–44], and additionally, are capable of driving plasticity post-SCI. Enhancing intrinsic growth capacity requires cell subtype-specific activation of growth as opposed to indiscriminate or off-target systemic treatment. Thus, additional studies will be needed to identify intrinsic modulators activated specifically in spared neurons after SCI.

The second obstacle to adult axon growth is the inhibitory extracellular environment of the CNS. Environmental inhibitors fit into 2 broad categories: myelin-associated inhibitors (MAIs) and the extracellular matrix-associated chondroitin sulfate proteoglycans (CSPGs). MAIs include NogoA [45–47], myelin-associated glycoprotein [48], and oligodendrocyte myelin glycoprotein [49]. These inhibitors impede neuronal growth by signaling through neuronal Nogo receptors 1 and 3 (NgR1/3) [49–52], PirB [53], PTPsigma [54, 55], leukocyte common antigen-related phosphatase [55, 56], and the sphingolipid receptor 2 [57]. Nullifying the effects of inhibitors by targeting MAIs and CSPGs either genetically or pharmacologically has led to encouraging but incomplete axon regeneration and functional recovery [28, 50, 58–62]. More recently, strategies that combine ≥ 1 monotherapies have shown promise but remain clinically unsatisfactory [22, 63]. Common to all experimental models of mild and severe SCI is the emergence of spontaneous recovery of function, despite the absence of axonal regeneration of the main descending motor pathway, the CST. As previously highlighted, an alternative mechanism supporting spontaneous recovery of function would be localized growth or plasticity of intact spinal circuitry. Accumulating data support this hypothesis as plasticity within several major descending motor tracts, particularly the CST, has been shown to directly mediate functional recovery after subcomplete SCI.

## Evidence Supporting a Functional Role for Spontaneous CST Reorganization

The CST is thought to be the primary descending motor tract in mammals in controlling voluntary movements [12, 13]. Evolutionarily, the CST has taken over the function of other vestigial tracts as fine motor movement and sophisticated motor planning became more important for complex animal behavior. In rodents, the CST runs in 3 white matter locations in the spinal cord, with most CST axons in the dorsal funiculus and, to a lesser extent, in the dorsolateral and ventral funiculi (summarized in Fig. 1). The CST is most commonly visualized using multiple stereotaxic injections of BDA into sensorimotor cortex. However, this approach is highly variable among investigators and labels 10% of the CST at best [11], which compromises our capacity to detect CST regeneration and/or plasticity after SCI. More comprehensive CST labeling using transgenic lines, including the *thy1*-STOP-YFP x *Emx1*-Cre line [9], *thy1*-YFP-H line [10], and *crym*-GFP line [11], offer superior insight into growth of intact and lesioned CST pathways.

In experimental SCI models, the CST has demonstrated a limited capacity to undergo regeneration with many believing that restoring function with CST regeneration a futile effort [64]. In DhX and contusion models, lateral and ventral CST axons are often spared, potentially allowing for sprouting and reorganization caudal to the lesion, although this mechanism has received limited investigation owing to insufficient CST labeling with BDA [65]. Lateral and ventral components of the CST only comprise 4% of the CST in mice [11]; therefore, insufficient and inconsistent labeling of the CST with BDA labeling makes these minor components difficult to study.

**Fig. 2:**
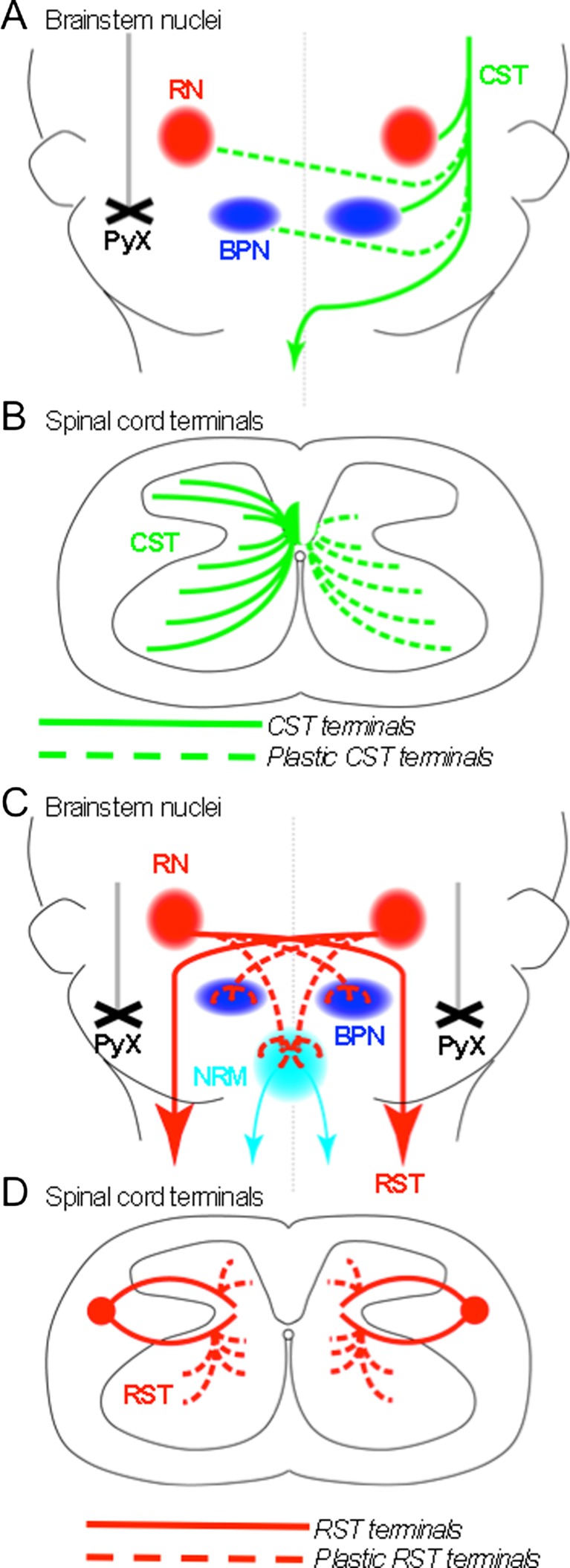
Schematic summary of anatomical plasticity of intact descending motor pathways after partial spinal cord injury. (**A**) The normal termination pattern of intact corticofugal circuitry (green lines) and lesion-induced *de novo* circuits (green stippled lines) after unilateral pyramidotomy (PyX; black cross). Corticofugal connections with the red nucleus (RN) and the basilar pontine nuclei (BPN) are unilateral in the intact adult; however, intact corticofugal neurons make bilateral connections after unilateral PyX. (**B**) After decussating in the caudal medulla, the majority of the corticospinal tract (CST) projects down the spinal cord in the ventral dorsal columns and terminates unilaterally within spinal gray matter (green lines). However, after unilateral PyX intact CST neurons sprout axons across the midline into denervated spinal territory (green stippled lines). (**C**) Normal path of the rubrospinal tract (RST) through the brainstem and *de novo* termination pattern or rubral circuitry (red stippled lines) after bilateral PyX (black crosses). In the intact adult, the RST does not make significant contact with the BPN or the nucleus raphe magnus (NRM); however, after complete bilateral destruction of CST, *de novo* rubral connections between the RN and the BPN and RN and NRM emerge. (**D**) After decussating in the tegmentum, the RST projects down the spinal cord in the lateral columns and terminates unilaterally within intermediate spinal lamina ( red lines). However, after bilateral PyX intact RST neurons sprout into more dorsal and ventral regions of the spinal cord (red stippled lines)

Despite numerous reports that the axotomized CST is unable to mount a significant long-distance regenerative response without intervention [5, 28, 59, 60], several studies have shown that the injured CST can undergo spontaneous short-distance sprouting after an incomplete SCI [4–7, 40]. Therefore, these more localized structural rearrangements may be substantially contributing to spontaneous recovery through direct contact with brainstem motor circuits or via relay connections through intact propriospinal circuits. For example, neonatal rats that underwent uPyX at postnatal day 2 (P2), a developmental time point during which the CST is still actively growing down the spinal cord [66], showed axon collaterals from the ipsilesional CST established bilateral corticorubral and corticopontine connections [67]. This *de novo* bilateral corticofugal circuit (summarized in Fig. 2A) was also shown to partially drive electromyographic (EMG) activity in contralesional denervated muscles post-injury, as microstimulation of the ipsilesional cortex resulted in bilateral EMG activity. This bilateral control of muscle activity was driven through lesion-induced corticorubral connections, as transient inactivation of the red nucleus using the γ-aminobutyric acid (GABA) receptor agonist muscimol eliminated the bilateral EMG activity.

In the above example, plasticity within corticorubral connections was driven by axotomy; however, axonal injury is not a requirement for the initiation of spontaneous CST sprouting after an incomplete SCI. Studies in perinatal mice (P7) have shown that intact spinal CST axons can sprout across the midline after contralateral CST injury (summarized in Fig. 2B) [5]. However, this plastic response is greatly diminished in the adult. The CST is able to mount a more robust growth response at P7 for 2 reasons: 1) the CST is still in an active developmental growth mode at P7 [66]; 2) many extracellular inhibitors, such as aggrecan and myelin-associated glycoprotein, and their receptors, such as NgR1, are not expressed during this period [68], thus allowing guidance cues and intrinsic growth regulators to drive the long distance growth and patterning of the CST. However, the limited plasticity that does occur in the intact adult CST has been shown to have significant functional implications. For instance, adult rats that underwent lateral hemisection showed that the ipsilesional cortex developed an electrical response to ipsilateral paw stimulation, as measured via cortical voltage-sensitive dye imaging [69]. In the rodent, sensory and motor cortices overlap, producing extensive sensorimotor feedback loops [69]. Therefore, increased limb representation as assessed through sensory activation in sensorimotor cortex corresponds to a change in CST limb representation. Anterograde and retrograde tracing showed that the mechanism supporting the emergence of ipsilateral cortical control of ipsilateral hindpaw function was mediated by the sprouting of intact CST axons into the denervated spinal cord caudal to the lesion site [70]. These data highlight the pivotal role of uninjured motor circuit plasticity in supporting functional recovery after trauma, suggesting that exploiting the mechanisms that drive spontaneous plasticity could greatly improve functional outcomes.

## Exploitation of Spontaneous CST Reorganization to Enhance Functional Recovery

Spontaneous sprouting of the uninjured CST after an incomplete SCI begs the questions of how do these neurons respond to injury without axotomy, and furthermore, how do these signals translate into cytoskeletal rearrangements that result in axon sprouting and/or plasticity? To investigate the intrinsic mechanisms, Bareyre et al. [71] completed differential gene expression analysis from microarrays on cervical spinal tissue ipsi or contralateral to a uPyX. They found that expression of growth factors insulin-like growth factor and brain-derived neurotrophic factor (BDNF) and their receptors insulin-like growth factor receptor and tropomyosin receptor kinase B (TrkB) were significantly elevated in the denervated side of the cervical spinal cord after uPyX. These studies suggest that intact CST axons are attracted to sprout across the midline by the increased availability of neurotrophic support. Supporting this hypothesis, a subsequent study demonstrated that delivery of small interfering RNA to knockdown BDNF specifically in the denervated cervical cord after unilateral sensorimotor cortex lesion resulted in a reduction in midline sprouting of the uninjured CST in the cervical cord. Furthermore, this reduction in midline CST sprouting significantly abrogated functional recovery in the forelimb pellet retrieval task in lesioned mice [72]. These data highlight the sensitivity of intact adult CST axons to growth factors; however, delivery of neurotrophins therapeutically requires caution as many spinal and primary afferent circuits express receptors to neurotrophic factors, which upon binding could result in maladaptive plasticity in the form of neuropathic pain and autonomic dysreflexia [73–76].

Additional efforts towards understanding the molecular events that drive growth in intact CST axons after partial SCI have focused upon exploiting genes expressed during developmental time points, when spontaneous CST sprouting is robust, versus adult time points, when spontaneous CST sprouting is minimal. It has been hypothesized that this is due, in part, to decreased mTOR activity in adult neurons. Immunohistochemical detection of phospho-s6, a marker commonly used as a readout of mTOR activity, in P7 and 2-month-old mice reveals that there is a significant reduction in phospho-s6 in 2-month-old mice in sensorimotor cortex, indicating that mTOR activity decreases in CST neurons with age [5]. Reports exploring the growth potential of retinal ganglion cells after optic nerve crush injury have also shown that mTOR activity is required for neurons to undergo injury-induced regeneration [39]. To test the mTOR hypothesis in the CST, a potent inhibitor of the mTOR pathway, PTEN, was knocked out of CST neurons using a neonatal infusion of an adeno-associated virus–Cre into sensorimotor cortex of PTEN-floxed mice [5]. After uPyX, PTEN-floxed mice displayed robust midline sprouting of the uninjured CST, indicating that a reduction in mTOR activity may be a developmental switch that reduces the intrinsic growth capacity of CST neurons from perinatal development to adulthood [5]. In support of these data, a recent report showed that co-deletion of PTEN and suppressor of cytokine signaling 3 (SOCS3) specifically in intact CST neurons after uPyX resulted in extensive sprouting of intact CST axons across the spinal midline and restoration of skilled forelimb function [77]. These data confirm the therapeutic potential for specifically enhancing sprouting of spared neurons to establish functional connections.

The developmental switch that limits spontaneous sprouting of the CST is also due to the deposition of extracellular inhibitors and expression of their neuronal receptors during closure of development and/or adolescent critical periods [78–81]. Indeed, elimination of the extracellular inhibition in adult mice also drives CST sprouting in incomplete models of SCI. To eliminate CSPG-mediated inhibition, the bacterial enzyme chondroitinase ABC (ChABC) was delivered to adult mice after uPyX via bolus injection every other day for 10 days post-lesion through an intracerebroventricular cannula. ChABC treatment leads to the degradation of inhibitory glycosaminoglycan side chains from chondroitin sulfate core proteins [38], thus creating a more permissive environment for axon growth after SCI in adult animals [60, 82, 83]. ChABC-treated mice showed an increase in midline crossing of the intact CST into the denervated cervical cord, which was correlated with a significant improvement in impaired limb function [6].

Additionally, pharmacological targeting of myelin-associated inhibition also drives sprouting of intact CST axons after incomplete SCI. The monoclonal antibody IN-1, developed against an antigrowth agent in CNS myelin [84], later identified as NogoA [45–47], stimulates regeneration and plasticity in many CNS injury models [85]. Rats treated with IN-1 after uPyX showed an increase in corticorubral, corticopontine, and corticospinal fibers that crossed the midline, establishing bilateral corticofugal and corticospinal circuits. These animals also showed significant improvement in forelimb pellet retrieval, grid walking, and rope climbing tasks 6 weeks post-lesion [7]. In support of these studies, mice null mutant for the myelin-associated inhibitor NogoA and its receptor, NgR1, also showed enhanced CST midline sprouting and recovery of forelimb function after uPyX [4].

Activity within CNS neurons is crucial to refining circuits during development, adolescence, and in the adult during learning and memory [86, 87]. Activity within intact CST neurons has also been shown to drive sprouting after uPyX. Carmel et al. [88, 89] and Brus-Ramer et al. [90] showed that electrical stimulation of the intact motor cortex or intact pyramidal tract after contralateral uPyX resulted in a significant increase in CST midline sprouting after uPyX. Efforts to understand the molecular mechanisms underlying this effect are ongoing, but electrical stimulation of spared tissue after incomplete SCI may provide significant functional benefit for human patients with SCI. For example, deep brain stimulation of spared reticulospinal axons after spinal transection in adult rats resulted in significant functional improvement [91].

Together, these data clearly demonstrate that despite its limited capacity to regenerate after axotomy, the intact CST is capable of mounting a functionally significant growth response after partial SCI. Additional studies are required to identify and ultimately exploit the molecular mechanisms that drive spontaneous CST axon growth and furthermore determine whether these targets can be translated to enhance growth of other intact and damaged tracts. While much attention has focused on the CST, emerging data suggest that other intact descending motor tracts are also capable of functional sprouting after partial SCI.

## Evidence Supporting the Functional Capacity of RST Reorganization

The RST is thought to be functionally redundant to the CST and its evolutionary predecessor in controlling voluntary movement [14–16, 92]. The RST drives gross motor functions that do not require precision but retains the capability of controlling fine movements in the absence of the CST. This redundancy is especially apparent in rodents when removal of the CST either by bPyX or dorsal column crush results in only subtle impairments [17], while removal of both the CST and RST via a mid thoracic DhX results in hindlimb paralysis [93]. Thus, functional redundancy allows for the potential for RST terminal plasticity to restore CST circuit deficits and function after selective CST injury.

Complete and specific elimination of the CST via bPyX reveals the extent of plasticity of the RST to compensate for CST function and anatomical terminal territory (summarized in Fig. 2C, D). In unlesioned mice, the RST primarily innervates intermediate laminae of spinal gray matter with few RST terminals extending into the spinal ventral horn. Six weeks after bPyX, anterogradely traced RST projections expand into both the spinal dorsal and ventral horns in comparison to sham lesioned controls (Fig. 2D). Sprouting of intact RST fibers was enhanced in *ngr1*^*-/-*^ mice [17]. A previous study reported that *ngr1*^*-/-*^ mice also showed increased sprouting of intact CST axons [4], confirming NgR1 as a potent therapeutic target to enhance plasticity of intact axons.

Pharmacologic blockade of myelin-associated inhibition using IN-1 antibody treatment also enhanced RST plasticity. After bPyX, IN-1-treated rats showed a 2-fold increase in gray matter collaterals emerging from the RST in the cervical cord [25, 26]. These collaterals also extended further into the spinal ventral horn than the typical innervation territory of untreated lesioned animals or sham lesioned controls, a phenotype reproduced in *ngr1*^*-/-*^ mice after bPyX [17]. After bPyX, IN-1-treated rats demonstrated significant improvement in forelimb grasping tasks. Furthermore, electrical stimulation of the motor cortex after bPyX in IN-1-treated rats elicited an EMG response in contralateral forelimb muscles and, to a lesser extent, ipsilateral forelimb muscles [27]. The RST mediated this functional re-innervation of muscles, as infusion of the GABA receptor agonist muscimol into the red nucleus eliminated the cortically evoked EMG responses, thus confirming the functional potential for RST re-wiring.

The RST also exhibits rewiring in the brainstem after CST elimination, providing for additional loci to mediate recovery. Six weeks after bPyX, anterogradely traced red nucleus projections increased into both the ipsilateral and contralateral basilar pontine nuclei in lesioned animals in comparison with sham controls (Fig. 2C). These rubropontine projections were further enhanced after bPyX in *ngr1*^*-/-*^ mice [17]. Additionally, rubrofugal projections were also observed to increase after bPyX in the NRM. The rubro-raphe projection increase was also enhanced after bPyX in *ngr1*^*-/-*^ mice (Fig. 2C). Despite significant reorganization of the RST in the spinal cord and into the NRM, no significant difference in RpST termination in the spinal cord was observed after bPyX [17]. This suggests that if the RpST is mediating some functional recovery after injury it is likely due to increased innervation of the NRM from rubrofugal or corticofugal projections. This was confirmed via transient inactivation of the NRM using virally delivered inhibitory DREADD receptors (designer receptor exclusively activated by designer drugs) [94]. Activation of the inhibitory DREADD receptor hM4di via acute delivery of clozapine-n-oxide in unlesioned animals resulted in no impairment in the grid-walking behavioral task. However, after bPyX, activation of hM4di resulted in abrogation of the incomplete functional recovery in the grid-walking task [17]. The transient loss in functional recovery was enhanced in *ngr1*^*-/-*^ mice, which had also shown increased rubro-raphe connections. These functional data demonstrates that the rubro-raphe connections are mediating a portion of the functional recovery after CST elimination.

## A Functional Role for Structural Plasticity Within Additional Descending Motor Tracts

Although the RST and CST redundantly control the majority of motor function in mammals, additional descending motor pathways play important modulatory roles in controlling motor function (see Fig. 1). This includes the RpST and the RtST. The RpST originates from the NRM and descends in the lateral funiculus to innervate the dorsal gray matter at all levels of the spinal cord [95]. Some species also contain a ventral RpST component [96], although it does not appear to modulate motor activity. The RpST is primarily known for its role in nociceptive modulation [97]; however, its connections with motor circuits in the spinal cord provide the potential for the RpST to act as a detour circuit to restore motor function after SCI [96]. In line with its role as a modulatory descending pathway, RpST neurons express the neuromodulator serotonin (5-hydroxytryptamine; 5-HT). The raphe nuclei are the primary source of 5-HT in the CNS, allowing for projections from the raphe to be visualized using immunostaining with 5-HT antibodies. Because an extrinsic or transgenic marker is not required to trace the RpST, it is relatively easy to study regeneration and sprouting of the RpST after SCI. This ease of RpST visualization has resulted in reports showing sprouting of potentially intact RpST axons in the ventral horn of the lumbar cord after SCI that correlate with hindlimb functional recovery [28, 59]. Evidence supporting a direct role for the sprouting of intact RpST axons in mediating functional recovery comes from selective pharmacological lesion studies using the serotonergic neurotoxin 5,7-dihydroxytryptamine [28]. Kim et al. [28] observed significant functional recovery and sprouting of RpST axons after complete transection in *ngr1*^-/-^ mice. However, delivery of 5,7-dihydroxytryptamine permanently abrogated the observed functional recovery suggesting that the sprouting of RpST terminals were crucial to post-injury recovery.

The RtST functions to control medial trunk muscles for postural support and movement preparation along with modulation of sensory and autonomic functions [98]. The RtST is comprised of a medial and lateral component both arising primarily from the medial reticular formation that descend near the medial longitudinal fasciculus and into the spinal cord (see Fig. 1). Both RtST components run in the ventral white matter of the spinal cord in the medial and lateral ventral white matter, respectively. The RtST projections are primarily ipsilateral with a small contralateral component. Visualization of the RtST is achieved via anterograde tracer injection [98]. Evidence of spontaneous RtST reorganization has been seen after lateral hemisection in adult rats [29]. Retrograde tracers infused into the denervated spinal cord below the injury site revealed an increase in the number of cells labeled in the contralesional gigantocellular reticular nucleus. To label the RtST projections after lateral hemisection in the rat, an anterograde tracer was injected into the gigantocellular reticular nucleus and revealed an increase in midline-crossing of RtST axons into the denervated cord at all levels of the spinal cord [29]. To investigate the functional implication of RtST reorganization, the gigantocellular reticular nucleus was acutely ablated by electrolytic microlesions in chronically injured lateral hemisected rats. Uninjured rats with reticular lesions showed no functional impairments; however, ipsi- or contrareticular lesions resulted in minor functional impairments in rats with chronic lateral hemisection injuries. These data demonstrate that tracts with only modulatory or limited direct effects on locomotion in an intact adult animal are able to contribute to functional recovery after injury due to spontaneous reorganization.

## The Next Phase

It is clear from the studies reviewed here that rebuilding a functional CNS after SCI does not require the recapitulation of long-distance axonal pathways that were established during development. Regeneration of damaged pathways and plasticity of intact circuits form a spectrum of axon growth that is crucial to functional recovery after SCI. Engaging intermediate circuits via localized sprouting of intact and lesioned axons onto propriospinal pathways (9, 23, 24) and plasticity of intact fibers that arborize into denervated territories (3-7, 40) are now established to have significant functional benefits [3–7, 9, 23, 24, 40]. The current challenge remains to more comprehensively map the post-injury *de novo* circuits that drive functional recovery. However, the next phase of investigation will focus the molecular signals that underlie plasticity of intact axons. To date this mechanism is unknown; however, several hypotheses are emerging that remain to be fully explored [5, 72]. For instance, loss of innervation to interneurons and motor neurons in the spinal cord results in disruption of electrical signals within local spinal circuits. Changing the firing pattern within a spinal segment may induce anatomic changes to occur through classical synaptic plasticity or developmental pruning mechanisms facilitated through sprouting of axonal branches, changes in dendritic spine dynamics, or activation of previously silent synapses. Additionally, loss of innervation may also result in these target neurons releasing a neurotrophic factor that would promote axon growth. As discussed earlier, CST neurons express TrkB and can respond to BDNF increases in the denervated spinal cord. It is unclear whether this is the only signal to promote CST sprouting and it is also unknown if the other spinal tracts express BDNF receptors to promote non-CST sprouting. In addition, CST and RST sprouting into brainstem nuclei establishes bilateral *de novo* circuits with neurons that have not lost innervation so is it is unlikely to be facilitated through the same mechanism. Targeting BDNF and TrkB as a means of improving recovery after SCI also risks increasing neuropathic pain, necessitating the discovery of additional and specific targets to restore motor function after injury.

In the development of motor systems especially the CST, both extrinsic and intrinsic factors are necessary to achieve a working corticospinal system. The extracellular environment needs to have guidance cues to direct and promote axon growth, and neurons need to be able to detect and respond to these cues and be able to amount a growth response. Therefore, there is likely a multifactorial molecular mechanism supporting sprouting of intact neurons in the adult CNS after injury. Understanding the cues that promote circuit reorganization and why only some neurons are able to mount a growth response will be crucial in designing therapies that can more fully utilize plasticity of intact circuits to fully realize their potential to restore function after acute and chronic SCI.

## Electronic supplementary material

Below is the link to the electronic supplementary material.

ESM 1 (PDF 1224 kb)
